# Soft robotic patterning of liquids

**DOI:** 10.1038/s41598-023-41755-5

**Published:** 2023-09-21

**Authors:** Giacomo Sasso, Nicola Pugno, James J. C. Busfield, Federico Carpi

**Affiliations:** 1 School of Engineering and Materials Science, Queen Mary University of London, Mile End Rd, London, E1 4NS UK; 2 Department of Civil, Environmental and Mechanical Engineering, University of Trento, Via Mesiano, 77, 38123 Trento, Italy; 3 Department of Industrial Engineering, University of Florence, Via di Santa Marta 3, 50139 Florence, Italy; 4 IRCCS Fondazione don Carlo Gnocchi ONLUS, Via di Scandicci 269, 50143 Florence, Italy

**Keywords:** Biomedical engineering, Mechanical engineering

## Abstract

Patterning of two or more liquids, either homogeneous in each phase or mixed with particles (including biological matter, such as cells and proteins), by controlling their flow dynamics, is relevant to several applications. Examples include dynamic spatial confinement of liquids in microfluidic systems (such as lab-on-a-chip and organ-on-a-chip devices) or structuring of polymers to modulate various properties (such as strength, conductivity, transparency and surface finishing). State-of-the-art strategies use various technologies, including positioners, shakers and acoustic actuators, which often combine limited versatility of mixing with significant inefficiency, energy consumption, and noise, as well as tendency to increase the temperature of the liquids. Here, we describe a new kind of robotic mixers of liquids, based on electro-responsive smart materials (dielectric elastomer actuators). We show for the first time how an efficient soft robotic device can be used to produce, via combinations of rotations and translations, various spatial patterns in liquids and maintain them stable for a few minutes. Moreover, we show that, as compared to a conventional orbital shaker, the new type of robotic device can mix liquids with a higher efficacy (~ 94% relative to ~ 80%, after 8 min of mixing) and with a significantly lower increase of the liquids’ temperature (+ 1 °C relative to + 5 °C, after 6 h of mixing). This is especially beneficial when mixing should occur according to controllable spatial features and should involve temperature-sensitive matter (such as biological cells, proteins, pre-polymers and other thermolabile molecules).

## Introduction

A broad range of systems and applications require an ability to manipulate and pattern liquids, either homogeneous in each phase or mixed with various kinds of particles (including biological matter, such as cells and proteins), by controlling their flow dynamics. One example is related to microfluidic systems, such as those for lab-on-a-chip or organ-on-a-chip devices, where fluidic control can facilitate specific interactions among cells and/or molecules^1,2^. Another example deals with polymer structuring, where fluidic control of reagents during polymerisation can be used to generate complex inner structures (including crystalline phases), affecting the final material properties (such as mechanical strength, electrical conductivity, optical transparency and surface finishing) and their possible anisotropy^3–5^. Yet another example is dynamic positioning of micro-/nano-particles or living cells into desired patterns (such as microarrays), as well as dynamic sorting, by means of fluidic control of liquids that carry them^6–8^.

Existing strategies for such manipulations and patterning of liquids use a variety of technologies, including positioners^9^, shakers^3–5^ and piezoelectric acoustic actuators^2,6,7^. They aim to achieve this through vibrations or movements generated by linear or rotary motors, combined to mechanical transmission components (cams, gears), which typically limit the versatility of mixing and make the system bulky, inefficient and noisy. Moreover, heat generated by the motors tends to increase the temperature of the liquids, challenging their mixing when they contain temperature-sensitive matter (such as biological cells, proteins, pre-polymers and other thermolabile molecules).

In order to overcome such limitations of state-of-the-art technologies, this paper describes a new robotic system based on smart materials, able to produce a combination of translations and rotations of a container for liquids. It is based on a particular kind of Electromechanically Active Polymers transducers^10^, known as Dielectric Elastomer Actuators (DEAs)^11–13^. In general, DEAs are able to convert electrical energy into mechanical work, by exploiting an electrostatically-induced compression of a soft electrically insulating membrane sandwiched between two compliant electrodes. Essentially, they can be thought of as rubbery capacitors, whose incompressible dielectric undergoes, upon electrical charging, a decrease in thickness and an expansion in surface, caused by the following Maxwell stress (for a planar actuator)^12^:

1 $$p= {\varepsilon }_{\mathrm{r}}{\varepsilon }_{0}{\left(\frac{V}{d}\right)}^{2}= {\varepsilon }_{\mathrm{r}}{\varepsilon }_{0}{E}^{2},$$

where $p$ is the electrostatic pressure exerted by the compliant electrodes on the elastomer, ${\varepsilon }_{r}$ is the relative permittivity of the dielectric material, ${\varepsilon }_{0}$ is the permittivity of vacuum, $V$ is the applied voltage, $d$ is the thickness of the dielectric layer and $E$ is the resulting electric field.

DEAs have already been used to create rotary movements from thin, soft and energetically efficient electro-responsive membranes. In particular, Anderson et al.^14^ demonstrated that a planar membrane coated with segmented electrodes could be sequentially activated to generate rotations. While that work was not intended for any specific use (as electromagnetic rotary motors remain the most effective solution), the concept and later developments from other groups^15,16^ showed the possibility of exploiting an electrical deformability of a soft, planar membrane in completely different ways.

In this work, we go beyond that approach, describing for the first time how a DEA-based lightweight and energy efficient structure can be used to produce, via combinations of rotations and translations, various spatial patterns in liquids and maintain them stable for a few minutes, with a high efficacy of mixing and a limited thermal impact on the samples.

**Figure 1:**
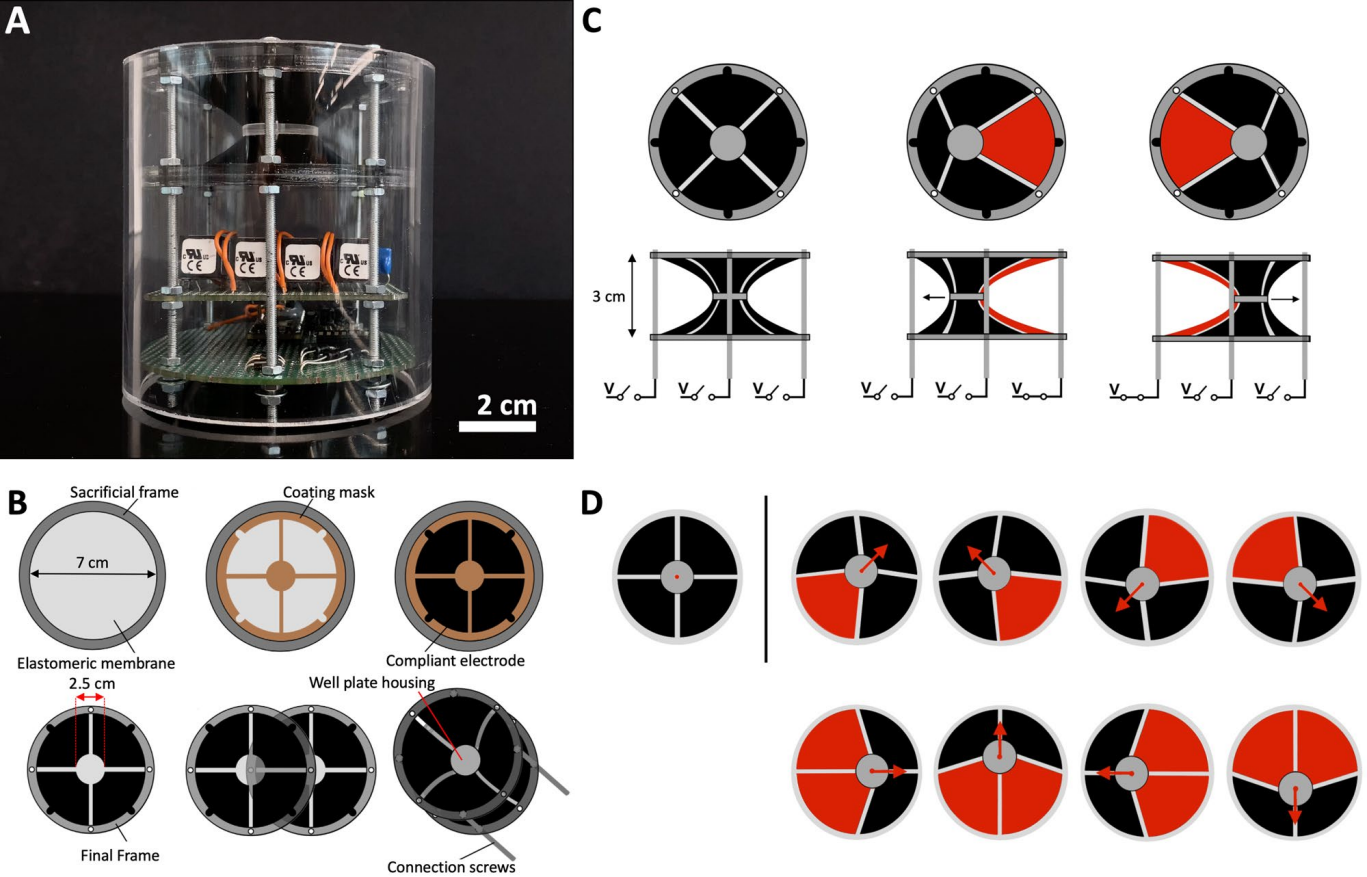
Structure, manufacturing and working principle of the DEA-based robotic mixer. (**A**) Photo of the device, showing the double-cone soft actuator (upper part) and its driving electronics (lower part), held in place by five screws, which were used to vary the actuator’s bi-axial pre-strain, so as to change its performance (see text). (**B**) Step-by-step manufacturing process of the double-cone soft actuator: an elastomeric membrane is radially pre-stretched and clamped to a circular frame; a coating mask is applied on it; a conductive material is deposited over the mask, producing four independent compliant electrodes; two electroded membranes are finally joined together at the centre, axially stretched (so as to achieve the double conical shape) and kept in place by the screws. Details are reported in “Methods” section. (**C**) Working principle of the device, schematically shown with top and side views; red colouring of an actuation sector indicates its electrical activation. (**D**) Examples of two activation sequences, where either one (top) or two (bottom) sectors are electrically activated at the same time.

In particular, the presented device is a new kind of robotic mixer of liquids, whose actuation was enabled by a so-called double-cone configuration of DEAs, consisting of two conically shaped elastomeric membranes, forming a 3D hyperbole^17–26^. That type of configuration has previously been shown to be effective for creating new types of soft actuators, possibly useful for robotic systems^17–26^. Here, we describe how it can be employed to generate controllable mixing patterns in liquids. Figure 1A shows a picture of the device.

As detailed in “Methods” section, each constitutive membrane was coated with a conductive material, forming four independent radial actuation electrodes on one side (schematically represented in Fig. 1B) and a continuous common ground electrode on the other side*.* The electrodes were deformable, so as to enable dielectric elastomer actuation. Each radial electrode in combination with the ground electrode formed an independent radial actuation sector. As a result, the whole structure consisted of eight actuation sectors, effectively corresponding to eight actuators (four for each cone). The device structure was kept in place by five long metal screws, which also served as electrical connections supplying the voltage signal to the four independent electrodes on each conical membrane and the common ground electrode. The screws also established an electrical connection between homologous electrodes (symmetrical about the well plate’s plane) on the two membranes, such that the corresponding two actuation sectors on the two membranes (one on each cone) acted in parallel. Accordingly, for simplicity from now on ‘sector’ will refer to a pair of homologous sectors activated in parallel.

The two electroded conical membranes were connected to a central end-effector, consisting of a well plate, where two (or more) liquids could be mixed, according to programmable mixing patterns, by modulating the electrical activation of the four actuation sectors. Indeed, the central well plate could be moved in different ways within its plane, by controlling the electrically induced expansion of each sector. In particular, by actuating one or more sectors at the same time, a linear displacement could be obtained (Fig. 1C), whilst sequential actuation of multiple sectors could generate complex motions within the plane (Fig. 1D).

The next sections present a characterisation of the performance of the device, as well as a demonstration of its ability to pattern liquids.

## Results

### Actuation performance

**Figure 2:**
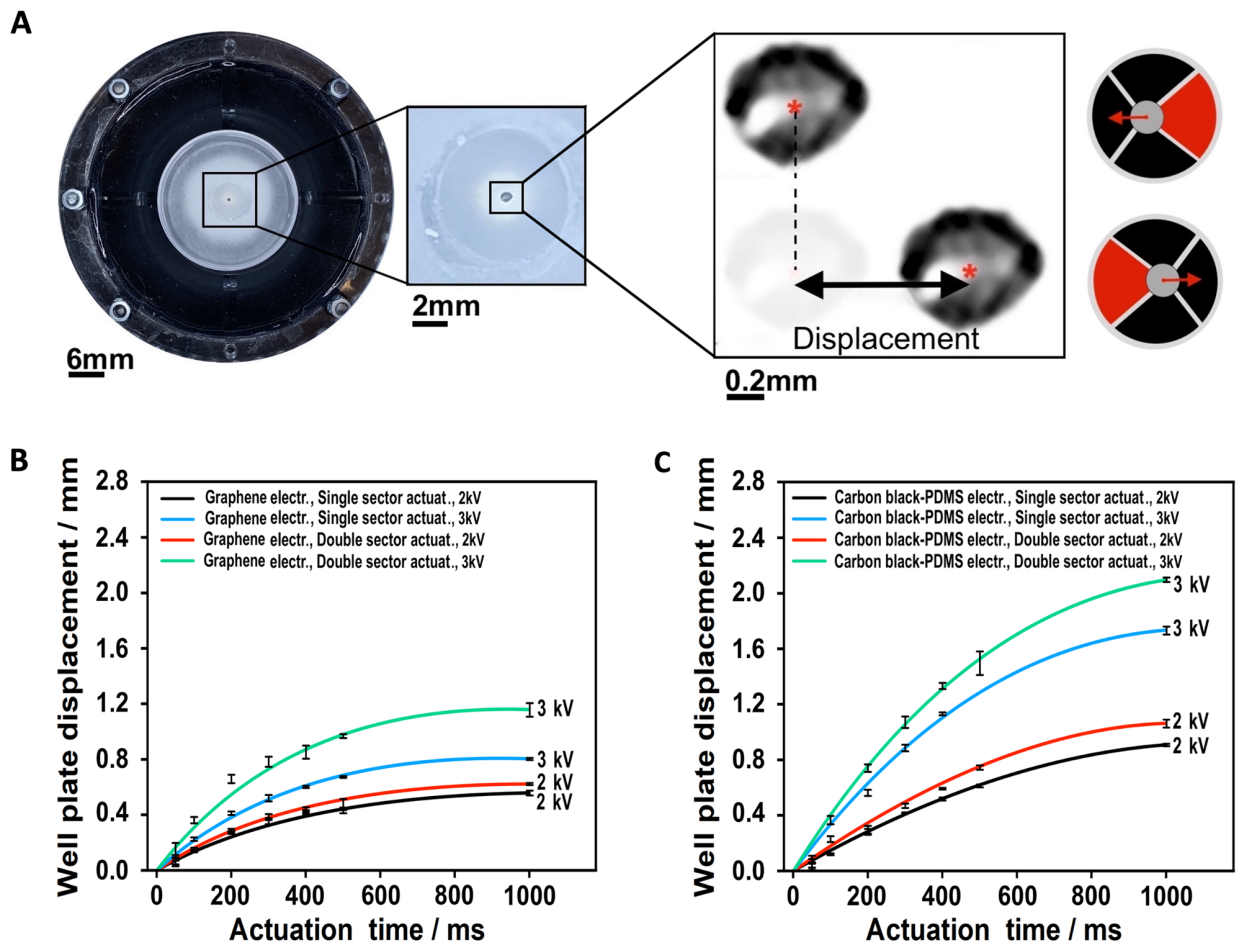
Actuation performance of the DEA-based robotic mixer. (**A**) Top view of the device, with a two-steps zoom-in on the well plate’s centre, where a marked dot was used to track the back-and-forth movement produced by the sequential activation of two diametrically opposite actuation sectors, as indicated on the right-hand-side schematic drawings; red colouring of an actuation sector indicates its electrical activation. (**B**) Well plate displacement as a function of different actuation times, for the device with graphene electrodes, and for single- and double-sector actuation. (**C**) Well plate displacement as a function of different actuation times, for the device with carbon black-loaded PDMS electrodes, and for single- and double-sector actuation. For all the data sets, the error bars represent the standard deviation among ten measurements. Fitting curves were added as a guide for the eye.

The device was characterised in terms of maximum displacement that could be induced on the well plate in response to step-wise voltage signals, consisting of a constant voltage applied for a given time (actuation time). In particular, the actuation time was tested in the range 50–1000 ms, at two actuation voltages: 3 kV (as an intensity ensuring an electric field safe for the pre-stretched membrane, preventing its electrical breakdown) and 2 kV (as a reduced voltage that was still able to produce macroscopic actuation). The voltage was sequentially applied to diametrically opposite actuation sectors, so as to create a back-and-forth motion of the well plate. The maximum displacement was measured as the difference between the two furthest positions reached, as detailed in “Methods” section. Figure 2A shows the experimental set-up.

Two different actuation modes were tested. In the first one (single sector actuation), only one actuation sector was activated at a time, generating a linear displacement in one direction, followed by a displacement in the opposite direction when activation was switched to the diametrically opposite sector. In the second mode (double sector actuation), two adjacent sectors were actuated simultaneously, generating a displacement (larger than that achieved with the single sector mode) in one direction, followed by a displacement in the opposite direction when activation was switched to the diametrically opposite sectors.

In order to investigate how different electrode materials were able to impact the actuation performance, these tests were performed on two versions of the robotic mixer, having compliant electrodes made of two different materials: a carbon black-loaded PDMS elastomer in one case, and graphene nanoplatelets in the other case. In their un-stretched state, the two materials had a sheet resistance of 516 kΩ/sq and 122 kΩ/sq, respectively. Details on composition and processing of these materials are presented in Methods.

The actuation performance measured on the two versions of the device is presented in Fig. 2B,C. As expected, for any combination of the parameters it was observed that a shorter actuation time caused a lower displacement, due to the viscoelasticity of the DEA membranes. Furthermore, for any electrode material, a double sector actuation produced a higher displacement relative to the single sector one, owing to the generation of a larger cross-sectional force. By comparing the performance, it is evident that the graphene electrodes (Fig. 2B) enabled significantly lower displacements relative to the carbon black-PDMS electrodes (Fig. 2C). This might be ascribable to a higher stiffness of the former. According to these outcomes, the carbon black-PDMS electrodes were selected as the preferred choice for the present application in order to perform the subsequent investigations described below.

### Mixing and thermal performance

**Figure 3:**
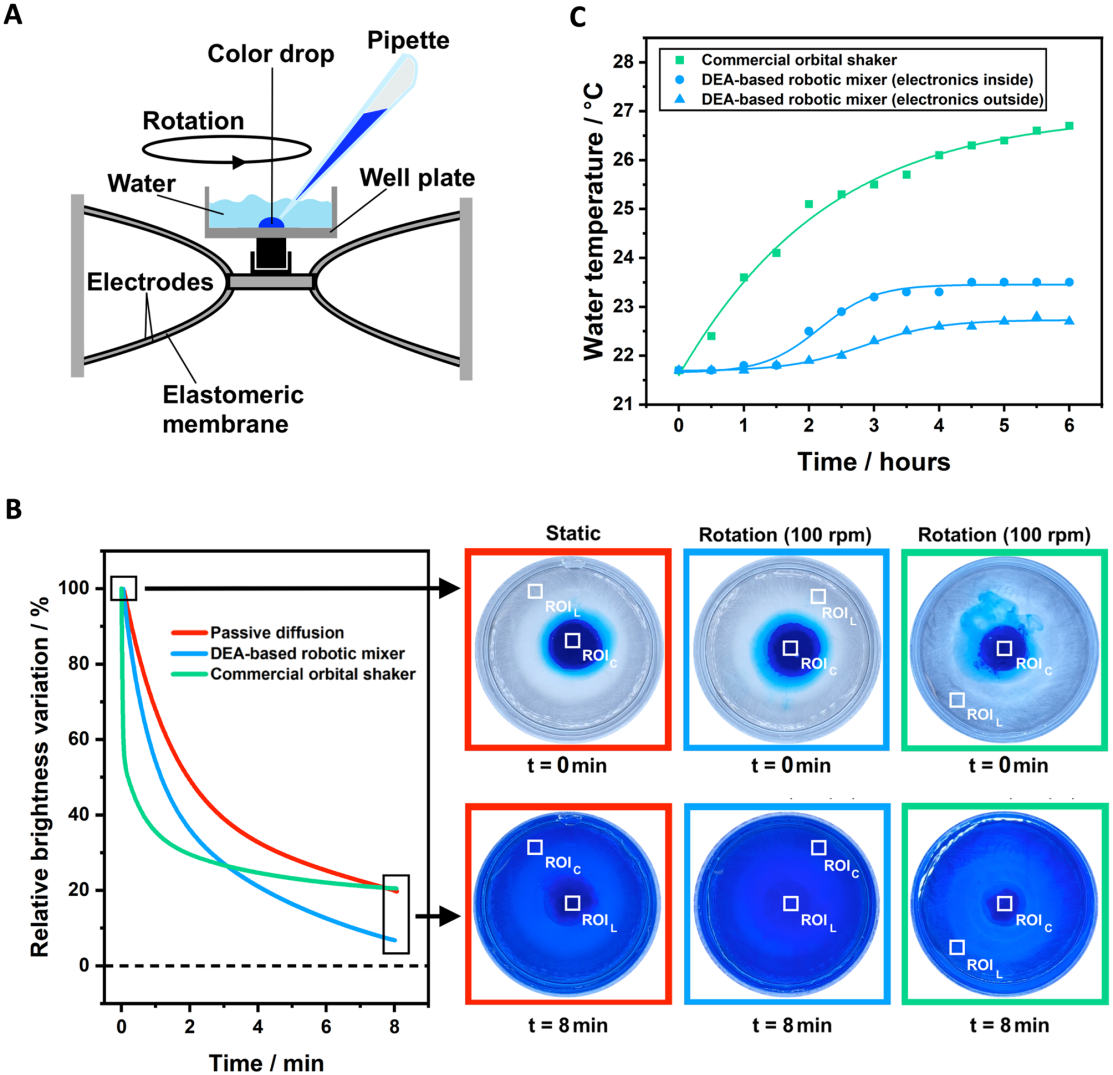
Mixing and thermal performance of the DEA-based robotic mixer. (**A**) Schematic drawing of the experimental setup for the mixing test with the DEA-based robotic mixer: after placing a drop of food colorant at the bottom of the well plate and submerging it in water, the mixer was operated to produce a circular motion at a rotational speed of 100 rpm. (**B**) Comparison of the fitting curves of the temporal evolution of the relative brightness variation during mixing, as enabled by the DEA-based robotic mixer (4.1% Root Mean Square Error RMSE fitting), the commercial orbital shaker (15.3% RMSE fitting) and static mixing (2.3% RMSE fitting); the images next to the plots show the actual mixing in the three cases, at the beginning of the process and after eight minutes. See Supplementary Movie 1. Although for the commercial orbital shaker the video recording accidentally started a few seconds after pouring water into the mixing container (as shown by the different initial condition relative to the other two cases), we point out that the slight delay did not have any significant effect on the subsequent measurements. (**C**) Comparison of the temporal evolution of the temperature of water inside the well plate moved with a circular motion by the commercial orbital shaker and the DEA-based robotic mixer with its high-voltage driving electronics either inside or outside the test chamber; polynomial fitting curves were added as a guide for the eye.

In order to investigate the mixing efficacy of the DEA-based robotic mixer, its performance was compared with that of a commercial orbital shaker (Orbital Shaker 1000, VWR International, USA), as well as with that of static mixing by simple passive diffusion. The maximum radius of the central plate’s circular motion for the DEA-based mixer was 1 mm, whilst that for the commercial orbital shaker was 7.2 mm. Comparative tests were performed as follows. A drop of a glucose-based food colorant (Food Colour Gel, Dr. Oetker, UK), whose measured density and viscosity were 1.3 g/cm^3^ and 5.7 Pa∙s, respectively, was initially placed at the centre of the well plate and then submerged in deionised water (Fig. 3A). For the passive diffusion-driven test, the well plate was maintained static. For the dynamic mixing tests with the DEA-based robotic mixer, the device was operated to create a circular motion at a rotational speed of 100 rpm. For the dynamic mixing tests with the commercial orbital shaker, the plate was lodged on the shaker and subject to a circular motion at the same rotational speed of 100 rpm. In each case, the temporal evolution of the mixing process was monitored using a video camera.

The mixing efficacy was evaluated via the following brightness-based greyscale image analysis. By considering the brightness value (from 0 to 255) of each pixel, a MatLab code was developed to measure the temporal variation of the average brightness inside two predefined regions of interest (ROI), which are shown in Fig. 3B for each setup. Each ROI had a significant number of pixels (100 × 100) to calculate an average brightness. The first ROI was selected from the plate’s lateral region (ROI_L_), where, at the beginning, no colorant was present. The second ROI was selected from the plate’s centre (ROI_C_), which contained the highest initial concentration of colorant. Therefore, those two ROIs had the highest initial difference in average brightness. For each video frame, the difference between the ROI_L_’s average brightness ${B}_{L}\left(t\right)$ and the ROI_C_’s average brightness ${B}_{C}\left(t\right)$ at the current time *t* was normalized by its initial value at *t* = 0 (at the start of the experiment), obtaining a relative brightness variation: $\left({B}_{L}\left(t\right)-{B}_{C}(t)\right)/\left({B}_{L}\left(0\right)-{B}_{C}(0)\right)$. The temporal change of the relative brightness variation was used as an indicator of the progression of the colorant’s dispersion in water, effectively measuring the extent of their mixing.

Results of these tests are presented in Fig. 3B, which plots the relative brightness variation as a function of time, in the three cases. Each curve corresponds to a data fitting with two exponential decays, whose dominant (smallest) time constant *τ* was as follows: *τ* = 0.03 min for dynamic mixing with the commercial orbital shaker, *τ* = 0.73 min for dynamic mixing with the DEA-based robotic mixer and *τ* = 1.42 min for static mixing.

As shown by the curves, the rotation applied in the two setups with dynamic mixing was able to facilitate the colorant’s dilution in water relative to static mixing, as expected. In particular, the commercial orbital shaker in the first few minutes performed better than the DEA-based robotic mixer, producing a faster drop in the relative brightness variation, likely due to the significantly larger radius of the well plate’s circular motion (7.2 mm for the former, as compared to 1 mm for the latter). However, the variation obtained with the commercial orbital shaker tended to stabilise, whilst a continuous drop was enabled by the DEA-based robotic mixer, such that the latter performed better after ~ 3 min (Fig. 3B). As a result, at the end of the observation time (8 min) the commercial orbital shaker lowered the relative brightness variation down to ~ 20%, whilst the DEA-based robotic mixer was more effective, as it was able to reach ~ 6%. By defining the mixing efficacy as $1-\left({B}_{L}\left(t\right)-{B}_{C}(t)\right)/\left({B}_{L}\left(0\right)-{B}_{C}(0)\right)$, such that it is null at *t* = 0 (for any initial difference of brightness) and it tends to 1 as differences in brightness tend to vanish, these results show that the efficacy at 8 min was ~ 80% for the commercial orbital shaker and ~ 94% for the DEA-based robotic mixer. This is also visible in the images presented in Fig. 3B, which show that at 8 min the DEA-based robotic mixer left a lower residual concentration of colorant at the well’s centre. The three experiments can be watched in Supplementary Movie 1. The video recordings show not only such a different mixing rate but also a different mixing modality. Indeed, whilst the DEA-based robotic mixer enabled a rather homogeneous dispersion, the commercial orbital shaker caused a sort of swirling motion of the colorant, which created periodic large variations of brightness at the Lateral ROI. This is reflected by the larger RMSE value that characterised the brightness data fitting for the commercial orbital shaker, as reported in the caption of Fig. 1.

The performance comparison between the DEA-based robotic mixer and the commercial orbital shaker was made also in terms of thermal effect that the operation of the device had on the liquid in the well plate. Indeed, heat production can be a key issue for any instrumentation when a temperature-controlled environment is required, in order to avoid thermal damage of sensitive matter contained in the liquids. As an example, this is the case of incubation of biological cell cultures inside heated adiabatic chambers, which have to keep a constant temperature of 37 °C for cellular survival. When a shaker/mixing device is used inside such chambers, heat produced by the motors can challenge the temperature control. In order to investigate such an effect, the DEA-based robotic mixer and the commercial orbital shaker were separately tested up to 6 h inside a cell-culture incubator (adiabatic chamber) with its own heating system turned off. The temperature rise that resulted from the operation of the mixing device alone was monitored using a thermocouple dipped into the water-filled well plate, while the device generated a circular motion of the plate, using the same rotational speed for both cases. Moreover, considering that for the DEA-based robotic mixer a significant heat source was expected to be represented by its high-voltage driving electronics, the mixer was tested in two conditions, differing for the location of the electronics: inside or outside the chamber. The latter case used thin high-voltage leads arranged through the incubator door seal.

Figure 3C presents the results of this thermal investigation, showing the temperature variation as a function of time for the three cases. As evident, the commercial orbital shaker produced a significantly higher temperature increase relative to the DEA-based robotic mixer. Moreover, the latter exhibited an even lower temperature variation when the electronics was located outside the chamber, as anticipated. In this case, the DEA-based robotic mixer caused a steady-state temperature increase of ~ 1 °C at 6 h, whilst the commercial orbital shaker produced a continuous rise, leading to an increase of ~ 5 °C at 6 h, without yet reaching equilibrium (Fig. 3C). This result is considered as particularly relevant, given that the commercial orbital shaker tested in this work is a type of system frequently used in biological and chemistry labs, in order to mix liquids that could contain biological cells, proteins, pre-polymers or any other temperature-sensitive molecule.

### Generation of complex motions of the well plate

By sequentially activating or deactivating different actuation sectors of the device, complex planar motions of the well plate could be achieved. The movement was video-recorded and tracked using a custom-made image processing code, as detailed in “Methods” section. By collecting the temporal evolution of the centre’s position on the plane, the path (trajectory) followed by the moving well plate was reconstructed and plotted on a blank image.

**Figure 4:**
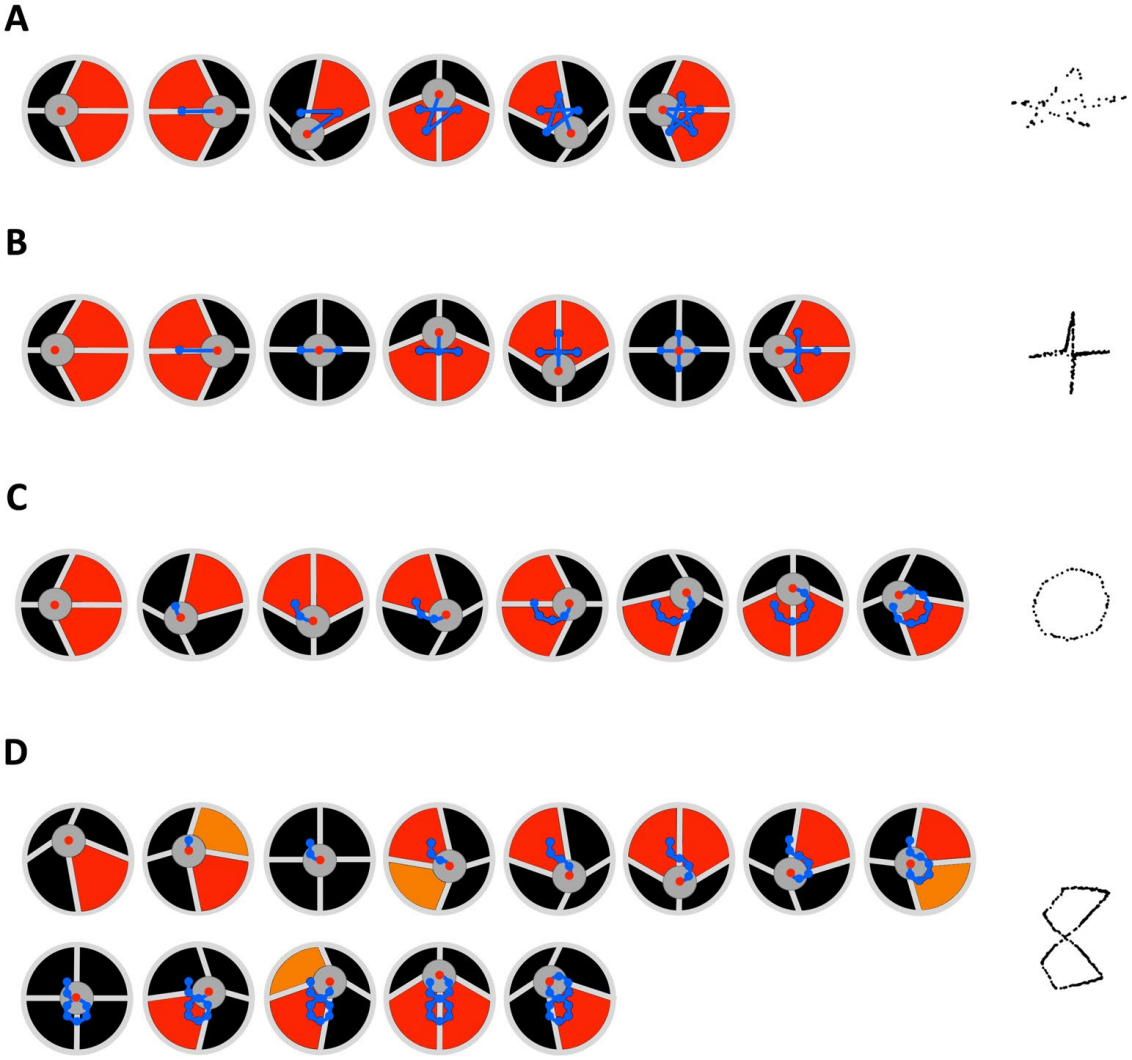
Generation of complex motions of the well plate with the DEA-based robotic mixer. Conceptual sequences of activation/deactivation of the actuation segments and corresponding paths followed by the well plate’s centre: (**A**) star; (**B**) cross; (**C**) circle; (**D**) figure of eight. Red and orange colouring of an actuation sector indicates electrical activation at 2 and 1 kV, respectively. The collection of blue dots in each drawing indicates the conceptual trajectory of the well plate’s centre up to that instant. The collection of tiny black dots on the right-hand side next to each sequence shows an example of an experimental trajectory. See Supplementary Movie 2.

Results are presented in Fig. 4, which shows for different paths (star, cross, circle and figure of eight) the corresponding sequence of electrical activation/deactivation of the actuation sectors. The experiments can be watched in Supplementary Movie 2.

The generated paths were centrally symmetric, having as a centre of symmetry the position of the well plate’s centre when none of the actuation sectors was activated. Each path was obtained by the combination of segments with different length and orientation, corresponding to sequential displacements along different directions, obtained by sequential activations/deactivations of the actuation segments. The length and orientation of the segments determined the accuracy of the actual path relative to the desired one, or, in other words, the resolution of the symbol represented by each path (star, cross, circle, figure of eight, in the examples of Fig. 4). The accuracy depended not only by the available degrees of freedom, which were limited by the number of independent actuation sectors, but also by the control variables for each sector, consisting not only in the applied voltage but also in the activation time. Indeed, if the elastomer were a purely elastic body, any given length of each segment forming the path (specifically, any given displacement) would be determined by the amount of electrical charge stored in the deformable capacitor (DEA), and, therefore, by the amplitude of the applied voltage. However, the viscous components of the elastomeric membrane made it a visco-(hyper-)elastic body, whose deformation depended not only by the voltage amplitude, but also upon the activation time, as also shown in Fig. 2.

### Spatial patterning of liquids

**Figure 5:**
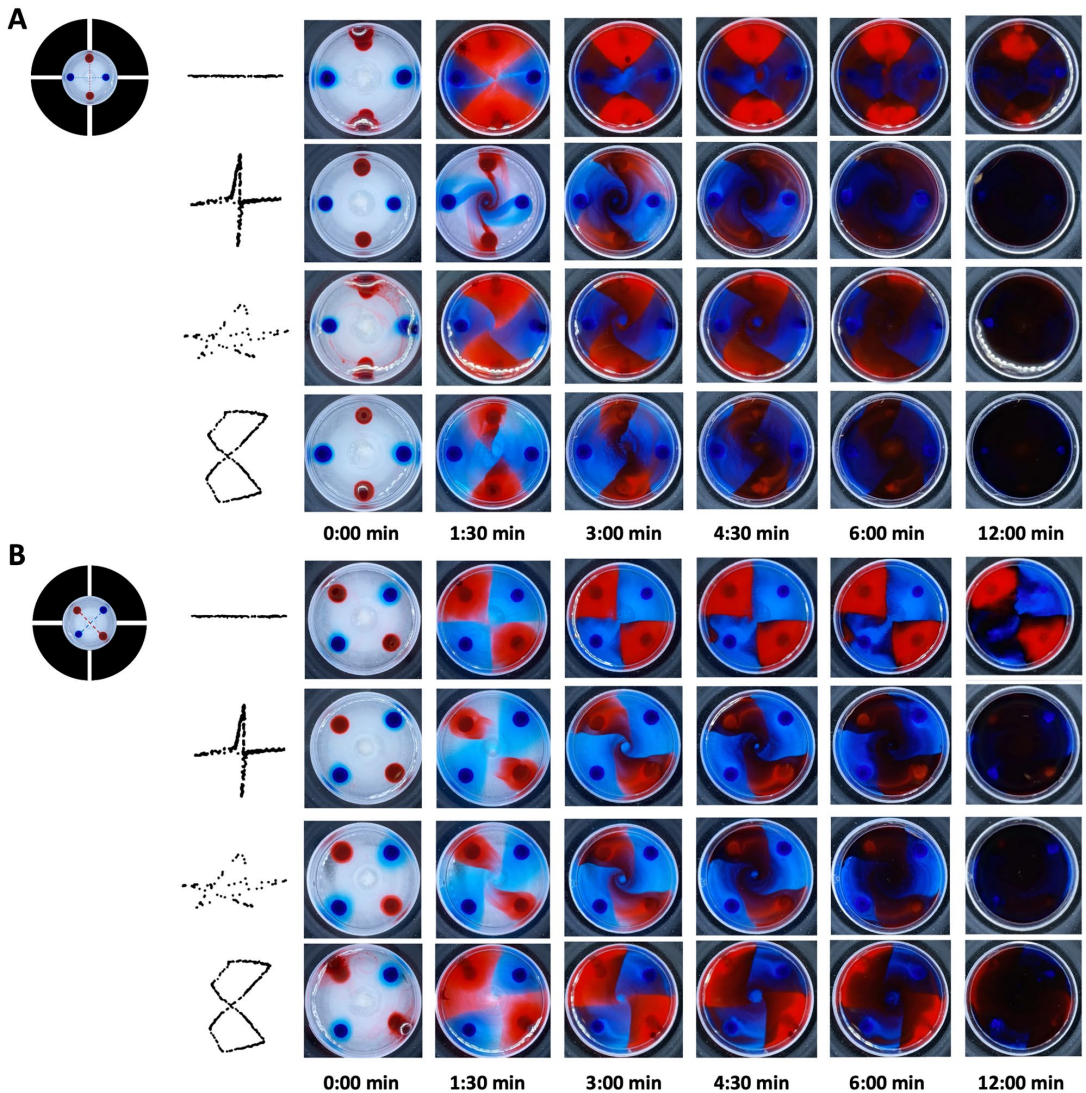
Spatial patterning of liquids by controlling their flow dynamics with the DEA-based robotic mixer. Comparison of the temporal evolution of mixing of two coloured water-based liquids inside the well plate, whose movement was controlled to follow a trajectory consisting of a segment, a cross, a star or a figure of eight, as represented on the left-hand side. The tests were performed for two configurations, differing for the initial position of the four drops of colorant: (**A**) positioned between every adjacent actuation sectors; (**B**) positioned at the midpoint of each actuation sector. See Supplementary Movie 3.

The complex motions of the well plate described above were then used to demonstrate the possibility of controlling the formation of various types of spatial patterns in liquids. In order to visualize the effect, four drops of a glucose-based food colorant were positioned at the bottom of the well plate, previously filled in with water. Two drops were red and two were blue. As shown in Fig. 5, each couple of drops of the same colour was arranged at diametrically opposite positions, close to the well’s edge, and the axes of the two couples were rotated by 90°. Moreover, two configurations were tested: in the first one, the four drops were positioned between two adjacent sectors (Fig. 5A), whilst, in the second one, the drops were positioned at the midpoint for each sector (Fig. 5B). The DEA-based robotic mixer was controlled to move the well plate along a path consisting of a segment, a cross, a star or a figure of eight, as described above. The resulting mixing dynamics of the fours drops of colorant in water were monitored using a video camera.

The results presented in Fig. 5 show the ability of the device to create different patterns and maintain them in a relatively stable formation, for a period of a few minutes. Interestingly, the duration of the stability of any given pattern was approximately doubled when it was created using the second configuration (Fig. 5B). The experiments can be watched in Supplementary Movie 3.

## Discussion

### Key innovative features

This paper described a soft robotic mixer of liquids, based on dielectric elastomer actuation, which made it possible to demonstrate complex mixing patterns. By varying the sequence of activation of the actuation sectors, as well as their actuation voltage and time, various kinds of mixing patterns could be produced and maintained stable for some minutes.

In comparison with a commercial shaker, this new robotic mixer could not only increase the versatility of mixing, enabling the generation of different mixing patterns (Fig. 5), but also mix more effectively in the long term (Fig. 3B) and with a lower production of heat (Fig. 3C).

The increased thermal protection of the sample under mixing was enabled by the electrostatic principle of operation of the actuation mechanism, as opposed to the electromagnetic technologies (electrical motors) employed in state-of-the-art shakers/mixers. This feature is important for applications that have to deal with temperature-sensitive fluids, such as those containing biological cells, proteins and pre-polymers.

Moreover, with regards to operations inside adiabatic chambers (such as an incubator for biological cell cultures), the new robotic mixer also offers a further facilitation: the possibility of arranging inside the chamber only the soft actuation part of the mixer, while displacing outside of it a significant heat source (represented in this case by the high-voltage driving electronics). This is not possible with conventional mixers/shakers, whose main heat source (represented by the electrical motors) should necessarily stay inside the chamber.

### Possible uses

Controllability of mixing dynamics in liquids is important for a wide range of possible applications. One example is dynamic spatial confinement of liquids in microfluidic systems, including lab-on-a-chip and organ-on-a-chip devices. This can satisfy diverse needs, such as promoting spatially and temporally selective interactions between an array of sensors and micro-/nano-particles or biological cells carried by a liquid, or, differently, promoting a controllable uptake by biological cells adhering to a substrate of nutrients carried by a liquid.

A different kind of use could be the creation of polymer-based items having a structuring of constitutive materials at various scales (from the macro-down to the submicron-scale). Depending on the targeted effect (which could include mechanical strength, surface texturing, colouring, etc.) and targeted resolution, different structuring strategies could be considered. For instance, in order to obtain graded elastic moduli, one could envisage a spatial and temporal confinement of polymerising liquid agents. As another example, in order to obtain a surface distribution of colours, various colorants could be mixed with a liquid UV-curable pre-polymer in a spatially controlled manner to obtain desired patterns, which then could permanently be stabilised by fast polymerisation with UV irradiation.

The last example was considered as a test case to demonstrate the potential of this new technology, by performing the following experiment aimed at obtaining a preliminary proof of feasibility. The setup shown in Fig. 5B was used to mix a clear UV fast curing resin with two pairs of red and blue coloured resin drops. The DEA-based robotic mixer’s control was such that the well plate had a linear motion (first case of Fig. 5B). As soon as the desired pattern was obtained, a UV lamp was used to stabilise it by fast curing of the resin. The resulting polymeric item is shown in Supplementary Fig. 2.

### Increase of performance

In order to increase the actuation performance of this device, as in general for any DEA, several material properties come into play, including the dielectric permittivity, the elastic modulus, the maximum strain that can be imposed before mechanical failure, the maximum electric field that can be sustained before dielectric breakdown (dielectric strength), as well as the viscoelastic behaviour, which determines the response speed.

Increasing the electrically induced strains requires a reduction of the elastic modulus and an increase of both the dielectric strength and dielectric permittivity, the latter being directly proportional to the generated Maxwell stress. This can be done by developing improved elastomeric formulations, although a trade-off among the different needs is typically unavoidable^27,28^.

Another factor that affects the actuation strains is represented by the membrane pre-strain. Indeed, while fabricating the device, the two membranes were bi-axially pre-stretched (see “Methods” section), in order to achieve, as for any DEA, a well-known increase in the electromechanical transduction performance, as first documented by Pelrine et al.^12^ and later on explained by Koh et al.^29^. In fact, pre-strain can enhance the dielectric strength and post-pone/suppress pull-in electromechanical instability^29^.

Moreover, in addition to the bi-axial pre-strain provided to the membranes during manufacturing, the device allowed for varying their bi-axial pre-strain also before operation. This was made possible by the five long screws visible in Fig. 1A, which carried nuts that could be moved along each screw to change the distance between the two ends of the device, effectively altering its height. The variable axial pre-tensioning of the device made the actuation performance tuneable, for any given applied voltage. Indeed, by increasing the height, the membranes’ pre-stretch increased and their thickness reduced, such that any given applied voltage could generate a higher electric field and therefore also a higher Maxwell stress. As a consequence, a higher pre-tensioning of the device was expected to induce a larger actuation of the well plate. In order to investigate such an effect, measurements of the maximum achievable displacement as a function of the actuation time were repeated, after an increase of its height from 3 to 4 cm. This provided each elastomeric membrane with an estimated increase of its final pre-stretch ratio from 3.46 to 3.78, corresponding to a decrease of its final thickness from 41.8 to 35 μm. Results, reported in Supplementary Fig. 3, confirmed that the higher pre-tensioning caused larger displacements of the well plate.

### High voltages: implications on safety, size and cost

The main limitation of this new mixing technology is the need for high driving voltages. This is certainly not desirable, especially for instrumentation that should be used by operators during routine lab testing and, in some cases, might also be employed to mix liquids containing particularly sensitive matter, such as biological cells. Nevertheless, this drawback in terms of electrical safety is mitigated by the two factors described below.

The first mitigation factor is the possibility of arranging the ground electrode on the upper face of the top membrane, so as to ensure protection for both the operator (in case of accidental contact) and any sensitive matter inside the well plate (such as biological cells). More generally, it is worth noting that DEAs have already been demonstrated to serve as a suitable technology to apply electrically controllable deformations to biological cell cultures adhering to stretchable substrates submerged in liquid media^30–34^.

The second mitigation factor is the possibility of driving the device at low electrical powers, as the electrical load is capacitive. The latter feature allows for using electrically safe power sources, such as the DC–DC voltage converters employed in this work, which could deliver a maximum power of only 0.5 W. The low power requirement also allows for the adoption of relatively compact high-voltage components, enabling the use of portable electronics. For instance, the DC–DC converter had the size of a 12.7 mm-sided cube.

Nevertheless, a further miniaturisation of the multi-channel high-voltage electronics would be desirable. One strategy for instance could be to implement a multiplexing of driving signals, through high-voltage transistors, from a single high-voltage source.

However, regardless of the strategy, the need for insulations will always make any kV-range electronics bulkier and more expensive (owing to a lower market share) than any electronics at one-order-of-magnitude lower voltages. Therefore, in order to make this technology more competitive, a reduction of the driving voltage to a few hundred Volts is highly desirable. This need can be addressed using the strategies discussed below.

### Reduction of the driving voltages

A reduction of the driving voltages should aim to reach the threshold of 500 V, as this is the range where highly compact electronics suitable to drive DEAs has been demonstrated^35^. Moreover, a few hundred Volts could advantageously be generated by low-size and low-cost electronics widely used for piezoelectric actuators. In order to lower the voltages, according to the Maxwell stress equation the following two strategies can be envisaged.

The first one deals with a long-term approach, consisting in the development of improved elastomers with a higher dielectric constant. Whilst this is feasible, the current challenge is to obtain durable materials, which, as pointed out above, should exhibit also a low elastic modulus and a high dielectric strength^27,28^.

The second strategy is more immediately applicable, as it deals with manufacturing soft elastomeric membranes with a lower thickness. For instance, silicone elastomers, even with off-the-shelf compositions, have been used to demonstrate the possibility of lowering the thickness of soft DEA membranes down to a few microns, with adequately low elastic moduli and high dielectric strength to ensure functional actuation^35,36^. Therefore, it is reasonable to envisage that DEA technologies in general might soon become usable with more compact and cost-effective electronics.

Nevertheless, membrane thinning is a strategy that implies the need for stacking multiple layers, so as to maintain sufficient elastic force. As a consequence, multi-layer manufacturing is expected to gain significant importance in the future.

### Expanding the controllability of the well plate motions

In this work, the well plate was moved along paths which maintained the initial position at rest as the centre of symmetry of the whole movement. Nevertheless, more complex movements could be achieved by combining a collection of paths having distinct centres of symmetry, with variable position on the plane. In other words, the symbols shown in Fig. 4 could be moved around and also combined in various sequences. This could be achieved by applying a variable offset voltage to one or multiple actuation sectors, so as to start creating any given path from different positions.

Moreover, out-of-plane movements could straightforwardly be achieved by activating the actuation sectors in different ways. Indeed, among the eight electrodes, in this work each pair of homologous electrodes on the two membranes was activated in parallel, such that the well plate could only have planar movements. Conversely, by de-coupling the electrodes, i.e. driving them independently, both vertical and tilting movements could be achieved, as shown in the preliminary test presented in Supplementary Movie 4. This would add more degrees of freedom for mixing in different ways and so creating different kinds of patterns.

## Methods

### Dielectric elastomer membrane

The device was manufactured using, as a dielectric elastomer membrane, a bi-adhesive acrylic-based elastomer film (VHB 4905, 3M, USA). The membranes were bi-axially pre-stretched, as specified in the following sections. They had an initial unstrained thickness of 0.5 mm, which then reduced to a lower value, depending on the applied pre-stretch, both during manufacturing (as detailed below) and before testing, by acting on the supporting screws (see the main text).

### Manufacturing of compliant electrodes made of a carbon black-loaded silicone

Compliant electrodes made of a conductive elastomer were obtained as a composite between a polydimethylsiloxane (PDMS) elastomer and a carbon black powder. The material was prepared according to the following steps. *Step 1* carbon black (Black Pearls 2000, Cabot, USA) was mixed with isopropanol solvent (Sigma Aldrich, UK), which was added with a ten-times-in-weight proportion, using a planetary mixer (THINKY ARE-250, Intertronics, UK) at a rotation speed of 2000 rpm for 2 min. *Step 2* the carbon black and isopropanol mixture was dispersed into a PDMS pre-polymer (MED4901, NuSil, USA) at a ratio of 9 wt%. *Step 3* an amount of isooctane solvent (2,2,4-Trimethylpentane, Sigma Aldrich, UK) equal to the amount of PDMS was added to the PDMS-carbon black mixture, and they were mixed together in the planetary mixer at 2000 rpm for 2 min, obtaining a fine dispersion of the carbon black into the PDMS. *Step 4* the resulting mixture was used to create compliant electrodes on the pre-stretched elastomeric membrane, by spray coating, using an airbrush. In order to ease spraying (reducing the likelihood of formation of clots and consequent intermittent flow), isopropanol solvent was added to the mixture right before filling in the airbrush reservoir. After spraying, the composite material was cured in an oven at 80 °C for 45 min, obtaining deformable electrodes on the elastomeric membrane’s surface.

### Manufacturing of compliant electrodes made of graphene

Compliant electrodes made of graphene nanoplatelets (GNPs—M25 Grade, XG Sciences, USA) were obtained by brushing the nanoplatelets on the pre-stretched elastomeric membrane. The tackiness of the membrane ensured adhesion, enabling the creation of a conductive network on the surface.

### Determination of the electrodes’ sheet resistance

The sheet resistance R_s_ of the electrode material was calculated from a measurement of resistance performed with an in-line four-point probe technique, where the diameter of the contacts was small relative to the probe distance (point contacts). By assuming an infinite sample, in such a configuration ${\mathrm{R}}_{\mathrm{S}}$ would be given by the following formula^37^:

2 $${R}_{S}=\frac{\pi }{\mathrm{ln}(2)}\frac{\Delta V}{I},$$

where $I$ is the applied current and $\Delta V$ is the measured voltage drop. However, for a finite sample, this formula should be modified according to the sample’s geometry. As our measurements were performed on circular samples, ${R}_{S}$ was calculated using the following relevant formula^38^:

3 $${R}_{S}= \frac{\pi }{\mathrm{ln}\left(2\right)+\mathrm{ln}\left(\frac{{D}^{2}}{{s}^{2}}+3\right)-\mathrm{ln}\left(\frac{{D}^{2}}{{s}^{2}}-3\right)}\frac{\Delta V}{I},$$

where *D* is the diameter of the sample (13 mm in our case) and *s* is the distance between the voltage probes (2 mm in our case).

### Manufacturing of the device

The double-cone soft actuator was manufactured according to the following steps. *Step 1* the dielectric elastomer membrane was equi-bi-axially pre-stretched by a factor of three (stretch ratio λ = 3), using a stretching tool. As a result of the pre-stretch, the membrane thickness was reduced from the initial value of 500 μm to the final value of 55.6 μm. In order to maintain the pre-stretch, the membrane was clamped to a pair of 3 mm-thick ring-shaped plastic frames, which provided the membrane with a free diameter of 70 mm (Fig. 1B). *Step 2* the membrane was masked, in order to prepare it for the deposition of the compliant electrodes. To that aim, a mask was obtained by LASER cutting the back sheet which the bi-adhesive dielectric elastomer membrane is coupled to and has to be removed before use. *Step 3* the mask, which enabled the creation of four radial sectors according to the shape shown in Fig. 1B, was attached to the pre-stretched adhesive dielectric elastomer membrane. *Step 4* the electrode material (described in the previous sections) was deposited on the masked membrane, so as to obtain the compliant electrodes. *Step 5* the mask was removed and the electroded membrane was clamped to a new circular frame. *Step 6* two electroded membranes were joined at the centre and axially stretched, in order to achieve the double-cone-shaped (3D hyperbole) device, as represented in Supplementary Fig. 1. Five screws were used to maintain the final shape of the structure, as well as to vary its bi-axial pre-strain, so as to change its performance (see the main text).

### High-voltage driving system

The actuation sectors were driven by high-voltage signals supplied by a custom-made, microcontroller-driven, four-channel, control unit. The unit included a micro-controller (Arduino Micro, Arduino, Italy), whose Pulse Width Modulated (PWM) channels were used to independently drive four DC-DC high-voltage converters (EMCO Q50, EMCO High Voltage, USA)—one for each actuation sector. Each converter was fed with a 0.7–4.0 V signal, to generate an output voltage up to 4 kV (the minimum input voltage of 0.7 V was found to be the lower limit of the converter’s linearity range). In order to generate the driving signal from each PWM signal, the latter was smoothed using a low-pass filter (made of a 4.7 µF capacitor and a 150 Ω resistor). In consideration of the high input current demand of each converter, its input was driven with a voltage follower (buffer), assembled with an integrated amplifier (TCA0372, ON Semiconductor, USA) and powered by a 5 V–1 A power supply. Moreover, the risk of back voltages was avoided by placing a Schottky diode in series to each converter’s input. In order to both let each converter operate with proper electrical loading and enable quick discharging of each actuation sector, a high-voltage discharge resistor of 200 MΩ was arranged in parallel to each converter’s output.

### Measurement of the electrically induced displacements

After drawing a dot at the well plate’s centre, the video-recorded movement of the dot was tracked using a MatLab-based custom motion tracking code, which processed each video frame as follows. Firstly, the image was cleaned, by using a combination of the MatLab morphological functions “erosion” and “dilation”. Secondly, the dot’s coordinates were detected using the MatLab connected-component-labelling function “regionprops”, which also printed on the image a red asterisk in coincidence with the dot. Thirdly, the variation of the coordinates was used to calculate the displacement.

### Measurement of the viscosity of the food colourant gel

A rotational shear test was performed by using a rheometer (DHR3 model, TA Instruments, USA) with a cone/plan geometry, having the following parameters: cone diameter 60 mm, cone angle 2°, shear rate sweep from 1 to 1000 s^−1^.

### Supplementary Information

Supplementary Information.

Supplementary Video 1.

Supplementary Video 2.

Supplementary Video 3.

Supplementary Video 4.

## Data Availability

The datasets used and/or analysed during the current study available from the corresponding author on reasonable request.

## References

[1] Wu Q, Liu J, Wang X (2020). Organ-on-a-chip: Recent breakthroughs and future prospects. Biomed. Eng. Online.

[2] Gu Y, Chen C, Mao Z (2021). Acoustofluidic centrifuge for nanoparticle enrichment and separation. Sci. Adv..

[3] Ibar J (1998). Control of polymer properties by melt vibration technology: A review. Polym. Eng. Sci..

[4] Fomin VN, Smolyaninov VV, Bobylev AP, Malyukova EB (2007). Effect of vibration on the structure and properties of polymer membranes. Biophysics.

[5] Ponomarenko A, Tameev A, Shevchenko V (2022). Action of mechanical forces on polymerization and polymers. Polymers.

[6] Vuillermet G, Gires P, Casset F (2016). Chladni patterns in a liquid at microscale. Phys. Rev. Lett..

[7] Glynne-Jones P, Hill M (2013). Acoustofluidics 23: Acoustic manipulation combined with other force fields. Lab Chip.

[8] Huang Z (2018). A general approach for fluid patterning and application in fabricating microdevices. Adv. Mater..

[9] Lu Z, Moraes C, Ye G, Simmons CA, Sun Y (2010). Single cell deposition and patterning with a robotic system. PLoS ONE.

[10] Carpi F (2016). Electromechanically Active Polymers: A Concise Reference.

[11] Carpi F, De Rossi D, Kornbluh R, Pelrine R, Sommer-Larsen P (2008). Dielectric Elastomers as Electromechanical Transducers: Fundamentals, Materials, Devices, Models and Applications of an Emerging Electroactive Polymer Technology.

[12] Pelrine R, Kornbluh R, Pei Q, Joseph J (2000). High-speed electrically actuated elastomers with strain greater than 100%. Science.

[13] Carpi F, Bauer S, De Rossi D (2010). Stretching dielectric elastomer performance. Science.

[14] Anderson IA (2010). A thin membrane artificial muscle rotary motor. Appl. Phys. A Mater. Sci. Process..

[15] Minaminosono A (2019). A deformable motor driven by dielectric elastomer actuators and flexible mechanisms. Front. Robot. AI.

[16] Waché R, McCarthy DN, Risse S, Kofod G (2015). Rotary motion achieved by new torsional dielectric elastomer actuators design. IEEE/ASME Trans. Mechatron..

[17] Li J, Wang Y, Liu L (2019). A biomimetic soft lens controlled by electrooculographic signal. Adv. Funct. Mater..

[18] Guo Y, Liu L, Liu Y, Leng J (2021). Antagonistic cone dielectric elastomer actuator: Analysis, experiment and application. Extrem. Mech. Lett..

[19] Conn AT, Rossiter J (2011). Antagonistic dielectric elastomer actuator for biologically-inspired robotics. Proc. SPIE Electroact. Polymer Actuators Devices.

[20] Branz F, Francesconi A (2016). Modelling and control of double-cone dielectric elastomer actuator. Smart Mater. Struct..

[21] Cao C, Gao X, Burgess S, Conn AT (2020). Power optimization of a conical dielectric elastomer actuator for resonant robotic systems. Extrem. Mech. Lett..

[22] Luan Y, Wang H, Zhu Y (2010). Design and implementation of cone dielectric elastomer actuator with double-slider mechanism. J. Bionic Eng..

[23] Zhang C, Sun W, Chen H (2016). Electromechanical deformation of conical dielectric elastomer actuator with hydrogel electrodes. J. Appl. Phys..

[24] Wang HM, Zhu JY, Ye KB (2009). Simulation, experimental evaluation and performance improvement of a cone dielectric elastomer actuator. J. Zhejiang Univ. Sci. A.

[25] Cao C, Chen L, Duan W (2021). On the mechanical power output comparisons of cone dielectric elastomer actuators. IEEE/ASME Trans. Mechatron..

[26] Cao C, Conn AT (2018). Performance optimization of a conical dielectric elastomer actuator. Actuators.

[27] Madsen FB, Daugaard AE, Hvilsted S, Skov AL (2016). The current state of silicone-based dielectric elastomer transducers. Macromol. Rapid Commun..

[28] Dünki SJ, Ko YS, Nüesch FA, Opris DM (2015). Self-repairable, high permittivity dielectric elastomers with large actuation strains at low electric fields. Adv. Funct. Mater..

[29] Koh SJA, Li T, Zhou J, Zhao X, Hong W, Zhu J, Suo Z (2011). Mechanisms of large actuation strain in dielectric elastomers. J. Polym. Sci. B.

[30] Imboden M, De Coulon E, Poulin A, Dellenbac C, Rosset S, Shea H, Rohr S (2019). High-speed mechano-active multielectrode array for investigating rapid stretch effects on cardiac tissue. Nat. Commun..

[31] Poulin A, Imboden M, Sorba F, Grazioli S, Martin-Olmos C, Rosset S, Shea H (2018). An ultra-fast mechanically active cell culture substrate. Sci. Rep..

[32] Poulin A, Saygili Demir C, Rosset S, Petrova TV, Shea H (2016). Dielectric elastomer actuator for mechanical loading of 2D cell cultures. Lab Chip.

[33] Costa J, Ghilardi M, Mamone V, Ferrari V, Busfield JJ, Ahluwalia A, Carpi F (2020). Bioreactor with electrically deformable curved membranes for mechanical stimulation of cell cultures. Front. Bioeng. Biotechnol..

[34] Cei D, Costa J, Gori G, Frediani G, Domenici C, Carpi F, Ahluwalia A (2016). A bioreactor with an electro-responsive elastomeric membrane for mimicking intestinal peristalsis. Bioinspir. Biomim..

[35] Ji X, Liu X, Cacucciolo V, Civet Y, El A, Cantin S, Perriard Y, Shea H (2020). Untethered feel-through haptics using 18-µm thick dielectric elastomer actuators. Adv. Funct. Mater..

[36] Poulin A, Rosset S, Shea H (2015). Printing low-voltage dielectric elastomer actuators. Appl. Phys. Lett..

[37] Miccoli I, Edler F, Pfnür H, Tegenkamp C (2015). The 100th anniversary of the four-point probe technique: The role of probe geometries in isotropic and anisotropic systems. J. Phys. Condens. Matter.

[38] Smits FM (1958). Measurement of sheet resistivities with the four-point probe. Bell Syst. Tech. J..

