# Supplementary material for: Soft robotic patterning of liquids
Source: Sci Rep. 2023 Sep 21;13:15739. doi: 10.1038/s41598-023-41755-5 (PMC10514051; doi:10.1038/s41598-023-41755-5)
Supplement: Supplementary file 1 — Supplementary Information. [file 41598_2023_41755_MOESM1_ESM.docx]

**Supplementary Information**

**Soft robotic patterning of liquids**

Giacomo Sasso, Nicola Pugno, James J.C. Busfield, Federico Carpi

**This PDF file includes:**

Figure 1. Schematic construction of the device.

Figure 2. Use of the technology to obtain a polymeric disc with spatial control of colours.

Figure 3. Increase of the actuation performance by increasing the axial pre-tensioning of the device.

**Other Supplementary Information includes the following:**

Movie 1. Mixing performance of the DEA-based robotic mixer in comparison with those of a commercial orbital shaker and passive diffusion.

Movie 2. Generation of complex motions of the well plate with the DEA-based robotic mixer.

Movie 3. Patterning of liquids by controlling their flow dynamics with the DEA-based robotic mixer.

Movie 4. Expansion of the controllability of the well plate motions.


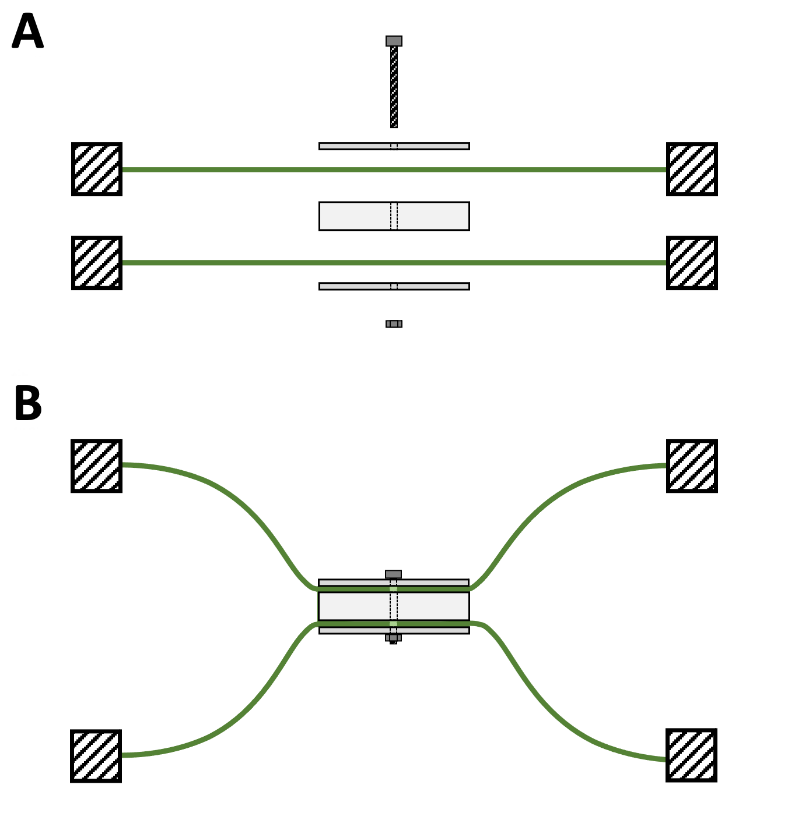


**Figure 1.** Schematic construction of the device: constitutive parts (**A**); assembled device (**B**).

**
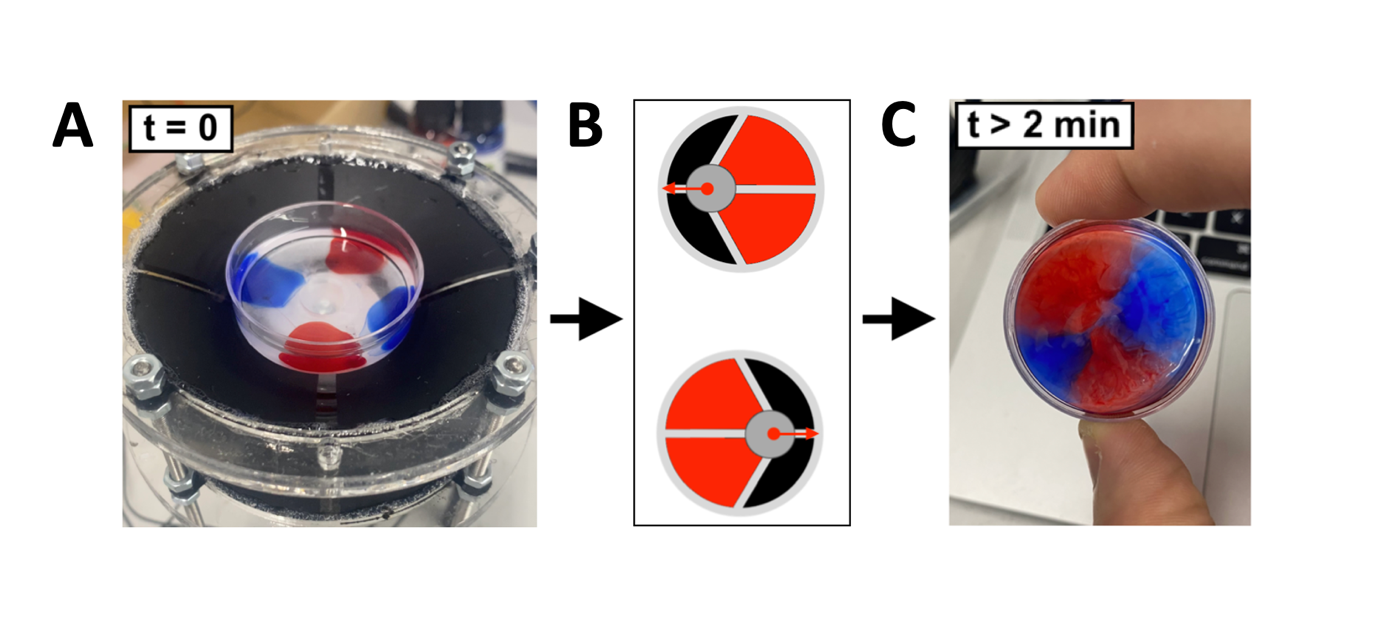
**

**Figure 2.** Use of the technology to obtain a polymeric disc with spatial control of colours. Red and blue coloured resin drops were put into a clear UV fast curing resin (**A**) and mixed with a linear motion of the well plate (**B**). Once the desired pattern was created, it was stabilised by quickly curing the resin with a UV lamp, obtaining a polymeric disc with coloured sectors (**C**).

**
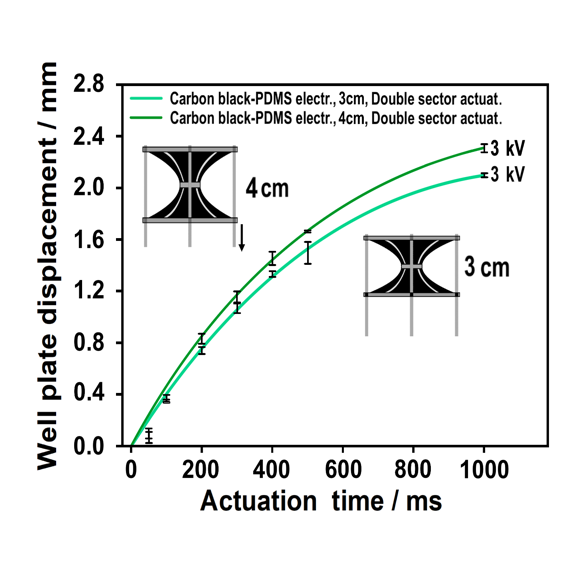
**

**Figure 3.** Increase of the actuation performance by increasing the axial pre-tensioning of the device. By displacing the lower end of the device along its screws, its height was extended from 3 to 4 cm. This caused on each membrane an estimated increase of its final pre-stretch ratio from 3.46 to 3.78, corresponding to a reduction of its final thickness from 41.8 to 35 μm. As a result, the same applied voltage was able to induce larger movements of the well plate, owing to an increase of the driving electric field, according to the Maxwell stress effect. The error bars represent the standard deviation among ten measurements. Fitting curves were added as a guide for the eye.
